# Patterns and Drivers of Antifungal Prescribing in Acute Leukemia: A Retrospective Cohort Study

**DOI:** 10.1093/ofid/ofae094

**Published:** 2024-03-01

**Authors:** Hamish Houston, Peter Dutey-Magni, Matthew Steel, Selina Patel, Wai Keong Wong, Laura Shallcross, Andrew James Wilson, Neil Stone

**Affiliations:** Department of Clinical Microbiology, University College London Hospitals NHS Foundation Trust, London, United Kingdom; Institute for Health Informatics, University College London, London, United Kingdom; Medical Research Council Clinical Trials Unit, University College London, London, United Kingdom; Clinical and Research Informatics Unit, University College London Hospitals NHS Foundation Trust, London, United Kingdom; Institute for Health Informatics, University College London, London, United Kingdom; Department of Haematology, University College London Hospitals NHS Foundation Trust, London, United Kingdom; Institute for Health Informatics, University College London, London, United Kingdom; Clinical and Research Informatics Unit, University College London Hospitals NHS Foundation Trust, London, United Kingdom; Department of Haematology, University College London Hospitals NHS Foundation Trust, London, United Kingdom; Institute for Health Informatics, University College London, London, United Kingdom; Clinical and Research Informatics Unit, University College London Hospitals NHS Foundation Trust, London, United Kingdom; Department of Clinical Microbiology, University College London Hospitals NHS Foundation Trust, London, United Kingdom

## Abstract

**Background:**

Patients with hematological malignancy are at high risk of invasive fungal infections (IFIs). Diagnosis is challenging, which can lead to overtreatment. Reducing exposure to inappropriate antifungal prescribing is likely to improve patient safety, but modifying prescribing behavior is difficult. We aimed to describe patterns and drivers of therapeutic antifungal prescribing in a large tertiary hemato-oncology center in the United Kingdom.

**Methods:**

We studied adults receiving treatment for acute leukemia at our center between 1 April 2019 and 14 October 2022. We developed a reproducible method to analyze routinely collected data on antifungal therapy episodes in a widely used electronic health record system. We report antifungal use in days of therapy stratified by level of diagnostic confidence, as defined by consensus diagnostic guidelines (European Organisation for Research and Treatment of Cancer/Mycoses Study Group).

**Results:**

Two hundred ninety-eight patients were included in the analysis; 21.7% of inpatient antifungal use occurred in cases of proven/probable IFI. Substantial antifungal use occurred in the absence of strong evidence of infection in patients receiving high-intensity first-line chemotherapy or approaching death (81.0% and 77.9%, respectively). Approximately 33% of high-resolution computed tomography (HRCT) reports were indeterminate for IFI. Indeterminate reports were around 8 times more likely to be followed by a new antifungal therapy episode than a negative report.

**Conclusions:**

Antifungal stewardship remains challenging in the absence of reliable diagnostics, particularly in more unwell patients. The proportion of antifungal therapy given for proven/probable infection is a new metric that will likely be useful to target antifungal stewardship programs. The thoracic HRCT report is an important contributor to diagnostic uncertainty.

## Teaser

A novel and reproducible antifungal consumption metric is demonstrated in this study of 298 patients with acute leukemia, of whom 41.6% received treatment for invasive fungal infection. Calculating the proportion of targeted antifungal therapy identified opportunities and challenges for antifungal stewardship.

## Graphical Abstract

**Graphical abstract:**
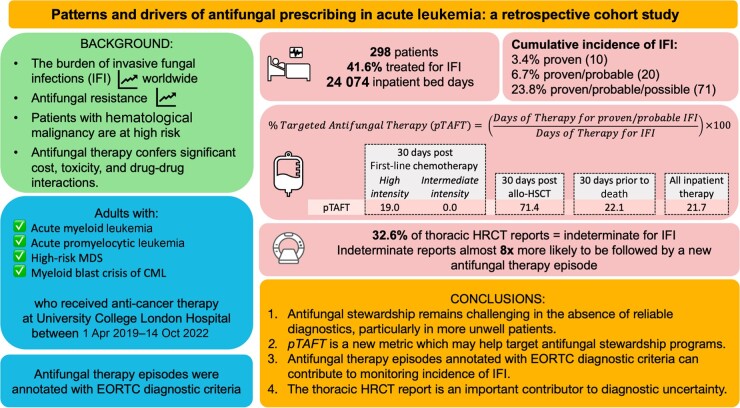

The burden of invasive fungal infections (IFIs) is increasing worldwide. The population at risk is growing as global access to an ever-increasing range of immunosuppressive therapies improves and the geographic range of fungal pathogens expands, in part due to climate change. However, IFIs remain difficult to diagnose and cause high morbidity and mortality [1]. Recognizing this growing threat, the World Health Organization published the first priority fungal pathogens list in 2022 [2, 3]. This increasing burden of disease is compounded by antifungal resistance, which weakens our limited arsenal of drugs effective against invasive infection [4, 5]. Injudicious use of these agents risks promoting antifungal resistance, as well as exposing patients to unnecessary toxicities.

The myeloid lineage, especially the neutrophil, is critical in the host response against IFI. Patients with hematological disorders affecting the myeloid line, including acute myeloid leukemia (AML) and high-risk myelodysplastic syndrome (HR-MDS), are at increased risk of IFI due to both their underlying disease and the immunosuppressive anticancer therapies used in their treatment. Proven diagnosis, which requires evidence of tissue invasion or recovery of fungus by culture of samples from a normally sterile site, is frequently not achieved. As such, diagnosis relies on surrogate markers of infection. Consensus diagnostic guidelines, published by the European Organisation for Research and Treatment of Cancer and the Mycoses Study Group (EORTC/MSG), categorize “probable” and “possible” IFI based on a combination of host, mycological, and clinical criteria [6].

Antifungal therapy has significant cost, toxicity, and drug–drug interactions. Therefore, identifying opportunities to reduce exposure to unnecessary prescribing is likely to improve patient safety. Understanding the patterns and drivers of inappropriate antimicrobial prescribing is a global health priority [7]. However, the majority of work to date has focused on use of antibiotics rather than antifungal drugs. Data are particularly limited in high-risk populations. A recent international Delphi survey established essential metrics for antifungal stewardship (AFS) programs, including antifungal use by indication [8]. However, several metrics deemed of high importance were given low feasibility rankings.

We aimed to describe patterns and drivers of therapeutic antifungal prescribing in a large tertiary hemato-oncology center in the United Kingdom (UK). We developed a reproducible method to monitor key antifungal usage metrics using data available from an advanced electronic health record system (EHRS). We annotated antifungal therapy episodes with EORTC/MSG diagnostic criteria for IFI to report antifungal use stratified by level of diagnostic confidence and facilitate estimation of incidence of IFI. We explored drivers of therapeutic antifungal prescribing in patients not meeting consensus diagnostic guidelines.

## METHODS

### Patient Cohort

We conducted a retrospective cohort study at University College London Hospital, one of Europe's largest centers for the treatment of hematological malignancies. We included all adults with AML, HR-MDS, acute promyelocytic leukemia (APML), and chronic myeloid leukemia in accelerated phase or blast crisis, who had received anticancer therapy and had at least 1 inpatient admission at our center between 1 April 2019 and 14 October 2022. Patients were excluded if they were <18 years old.

Eligible patients were identified contemporaneously by the acute leukemia team, and retrospectively by searching the regional Specialist Integrated Haematological Malignancy Diagnostic Service (SIHMDS) database (containing bone marrow and peripheral blood pathology reports) using the terms “AML,” “APML,” “acute leukemia,” “acute myeloid leukemia,” “therapy-related myeloid neoplasm,” and “chronic myeloid leukemia—blast crisis” [9].

### Data Sources

Details of hematological diagnoses were extracted from the SIHMDS database. Other data were collected from the EHRS (EPIC Systems, Verona, Wisconsin), including demographic information; inpatient episodes and *International Classification of Diseases, Tenth Revision* (*ICD-10*) codes; hematological treatment (chemotherapy and allogeneic hematopoietic stem cell transplantation [allo-HSCT]); antifungal treatment (prescription, administration, and dispensing records); body temperature and blood neutrophil count measurements; fungal biomarkers; mycological isolates; free-text histopathology; and thoracic high-resolution computed tomography (HRCT) reports.

### Antifungal Therapy Episodes

Prescription records for all systemic antifungal drugs were grouped into those given for treatment of fungal infection and those given for antifungal prophylaxis using structured indication data recorded at the time of prescribing. Consecutive or concurrent therapeutic antifungal prescriptions (prophylaxis excluded) were linked into therapy episodes using an R software package called Ramses (Resources for Antimicrobial Stewardship and Surveillance) [10, 11]. Consecutive prescriptions were deemed to be part of the same antifungal therapy episode if the time between the end of the previous prescription and the start of the subsequent prescription was <36 hours. Details of our centre's antifungal guidelines are provided in the Supplementary materials.

### Diagnostic Confidence

Antifungal therapy episodes were reviewed manually to identify those prescribed for treatment of IFI. Therapy episodes given for superficial infections (eg, oropharyngeal candidiasis) were excluded. Remaining therapy episodes were annotated using EORTC/MSG criteria (proven/probable/possible IFI) following a manual review of the case notes to determine level of diagnostic confidence [6].

### Measuring Antifungal Use

Antifungal use was measured using metrics that have been established by international consultation to be of high importance for AFS programs: days of therapy (DOT) adjusted for bed occupancy, DOT by antifungal class, and length of therapy (LOT) [8]. Recommended daily doses (RDDs) based on maintenance doses for treatment of IFI in the British National Formulary were also reported [12, 13]. Administration records (and dispensing records for analyses including outpatient usage) were aggregated by the level of diagnostic confidence of their associated therapy episode and used to measure antifungal use by indication. Antifungal use was estimated for proven, probable, and possible IFI as the proportion of all DOT, LOT, and RDDs prescribed to patients in each category. The proportion of targeted antifungal therapy (pTAFT) was calculated as shown in the following equation:


$$\begin{array}{rl} & pTAFT=\left(\frac{DOT\;for\;proven\;\&\;probable\;IFI}{DOT^{*}}\right)\times 100\\ & *prophylaxis\;excluded \end{array}$$

### Incidence of IFI

Therapy episode annotations were aggregated by patient. The maximum level of evidence of IFI reached in each patient was determined to estimate the cumulative incidence of IFI. All positive histopathology reports, fungal biomarkers, and mycological isolates not temporally linked to an antifungal therapy episode were reviewed manually to ensure no cases of IFI were missed.

### Episodes of Febrile Neutropenia

**Figure 1.**
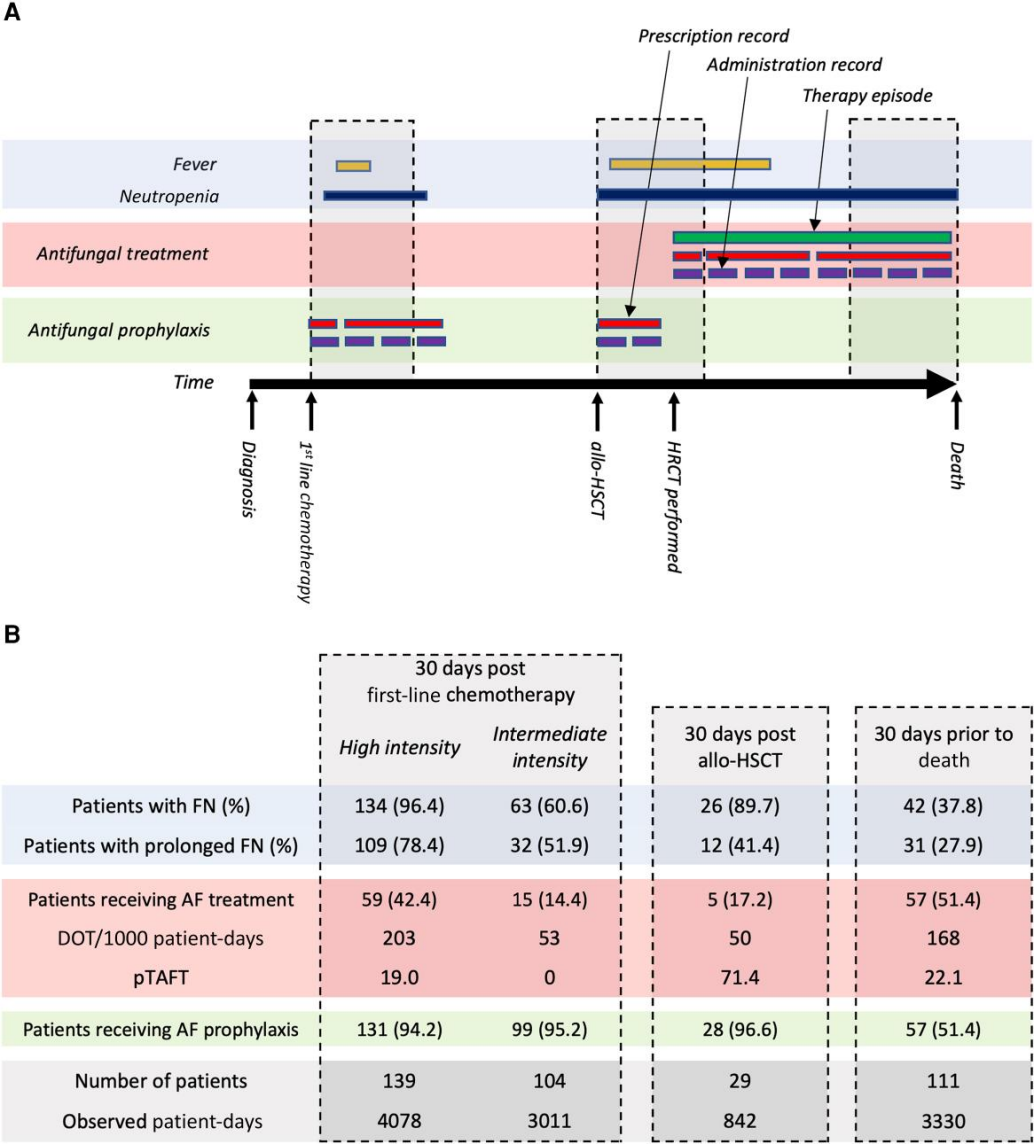
Antifungal therapy episodes and 30-day snapshots. *A*, The burden of febrile neutropenia was considered alongside antifungal use (inpatient and outpatient) during three 30-day snapshots: from day 1 of first-line chemotherapy, day 0 of allogeneic hematopoietic stem cell transplantation, and in the 30 days prior to death. First-line chemotherapy regimens were classed as high (containing anthracycline, fludarabine, or intermediate/high-dose Ara-C) or intermediate (containing venetoclax) intensity. *B*, Prescription records for all systemic antifungal drugs were grouped into those given for treatment of fungal infection and those given for antifungal prophylaxis using structured indication data recorded at the time of prescribing. Consecutive or concurrent therapeutic antifungal prescriptions were linked into therapy episodes using an R software package called Ramses (Resources for Antimicrobial Stewardship and Surveillance) [11]. Prescriptions for antifungal prophylaxis were excluded from these therapy episodes. Antifungal therapy episodes were annotated with a level of diagnostic confidence judged by European Organisation for Research and Treatment of Cancer/Mycoses Study Group criteria. Days of therapy were calculated using administration and dispensing records aggregated by level of diagnostic confidence of their associated therapy episode. Abbreviations: AF, antifungal; allo-HSCT, allogeneic hematopoietic stem cell transplantation; DOT, days of therapy; FN, febrile neutropenia; HRCT, high-resolution computed tomography; pTAFT, proportion of targeted antifungal therapy.

Onset of neutropenia was defined when neutrophil count dropped below 0.5 × 10^9^/L. Resolution of neutropenia was defined as an increase above this threshold unless recovery was sustained for <48 hours. Onset of febrile neutropenia was defined by a body temperature measurement >38.0°C occurring during a neutropenic period or within 48 hours of onset of neutropenia. Resolution of febrile neutropenia was defined by resolution of neutropenia or absence of fever for 48 hours. The relationship between the proportion of patients with febrile neutropenia and rate of antifungal use was examined within three 30-day snapshot periods (following day 1 of the first cycle of first-line chemotherapy, following day 0 of allo-HSCT, and prior to death; Figure 1*A*).

First-line chemotherapy regimens were classed as high intensity (containing anthracycline, fludarabine, or intermediate/high-dose Ara-C), intermediate intensity (containing venetoclax), low intensity (hypomethylating agent, low-dose Ara-C, or tyrosine kinase inhibitor without venetoclax), or arsenic containing.

### Thoracic High-Resolution Computed Tomography

Imaging reports viewed by researchers in the context of other clinical data (eg, antifungal prescribing data) may contribute to confirmation bias. Therefore, we conducted a blinded grading of imaging reports. Reports for all thoracic HRCT studies performed during the study period were extracted. Clinical information supplied in the imaging order was reviewed, to select studies performed for suspected respiratory infection, and was then redacted from the report. Studies performed while patients were receiving antifungal therapy, or shortly after a therapy episode had completed, were excluded. Reports were graded by author H. H. as “suggestive of IFI,” “indeterminate,” or “negative for IFI” as described previously [14]. The proportion of patients started on a new antifungal therapy episode within 48 hours of scan completion was reported.

### Statistical Methods

Descriptive data were summarized as proportional outcomes, means, or medians (ranges). Comparisons were made using χ^2^ tests of independence for categorical variables, and Wilcoxon rank-sum tests for nonnormally distributed numeric variables. Data extraction and statistical analyses were performed in R (R Core Team, 2020) within a trusted research environment.

## RESULTS

### Baseline Characteristics

Three hundred forty-two eligible patients were identified. Forty-four patients were excluded (5 were <18 years old, 26 had no inpatient admissions, and 13 received no treatment for leukemia at our center). A total of 298 patients were included and accumulated 24 074 inpatient bed-days. Median age of included patients was 64 years, 51% were female, and the median Charlson comorbidity score was 3 (Table 1).

**Table 1.** Cohort Demographics and Hematological Diagnoses

Characteristic	Overall (N = 298)	Not Treated for IFI (n = 174)	Treated for IFI (n = 124)	*P* Value	Treated Without Meeting Diagnostic Criteria (n = 53)	Treated for Possible IFI (n = 51)	Treated for Proven/Probable IFI (n = 20)
Sex							
Female	152 (51.0)	90 (51.7)	62 (50.0)	.86	26 (49.1)	25 (49.0)	11 (55.0)
Male	146 (49.0)	84 (48.3)	62 (50.0)		27 (50.9)	26 (51.0)	9 (45.0)
Age^a^, median (Q1, Q3)	64.3 (46.7, 73.6)	65.4 (47.2, 75.4)	62.6 (46.6, 71.4)	.0664	62.5 (46.4, 71.6)	62.6 (47.5, 71.2)	63.8 (48.9, 69.9)
Charlson comorbidity score^b^							
Median (Q1, Q3)	3 (2, 4)	3 (2, 4)	2.50 (2.00, 4.00)	.412	2 (2, 4)	3 (2, 4)	2.5 (2, 3.25)
Missing	7 (2.3)	5 (2.9)	2 (1.6)		0 (0)	2 (3.9)	0 (0)
Hematological diagnosis^c^							
AML, NOS	163 (54.7)	89 (51.1)	74 (59.7)	NA	34 (64.2)	31 (60.8)	9 (45.0)
AML with recurrent genetic abnormalities	41 (13.8)	21 (12.1)	20 (16.1)		8 (15.1)	9 (17.6)	3 (15.0)
AML with myelodysplasia-related changes	33 (11.1)	22 (12.6)	11 (8.9)		1 (1.9)	6 (11.8)	4 (20.0)
APML [AML with t(15,17)(q22;q12)], PML/RARA	26 (8.7)	22 (12.6)	4 (3.2)		1 (1.9)	0 (0)	3 (15.0)
Myelodysplastic syndromes	25 (8.4)	15 (8.6)	10 (8.1)		5 (9.4)	4 (7.8)	1 (5.0)
Myeloid blast crisis of chronic myeloid leukemia	7 (2.3)	4 (2.3)	3 (2.4)		2 (3.8)	1 (2.0)	0 (0)
Therapy-related myeloid neoplasms	2 (0.7)	1 (0.6)	1 (0.8)		1 (1.9)	0 (0)	0 (0)
Myeloid sarcoma	1 (0.3)	0 (0)	1 (0.8)		1 (1.9)	0 (0)	0 (0)
First-line chemotherapy^d^							
High intensity	143 (48.0)	62 (35.6)	81 (65.3)	<.001	33 (62.3)	37 (72.5)	11 (55.0)
Intermediate intensity	104 (34.9)	71 (40.8)	33 (26.6)		17 (32.1)	11 (21.6)	5 (25.0)
Low intensity	30 (10.1)	21 (12.1)	9 (7.3)		3 (5.7)	3 (5.9)	3 (15.0)
Arsenic containing	21 (7.0)	20 (11.5)	1 (0.8)		0 (0)	0 (0)	1 (5.0)
Follow-up^e^							
Deaths	111 (37.2)	57 (32.8)	54 (43.5)	.0755	23 (43.4)	21 (41.2)	10 (50.0)
Median days of follow-up (Q1, Q3)	562 (323, 969)	560 (396, 950)	563 (229, 1010)	.68	565 (276, 844)	631 (285, 1020)	551 (147, 1070)

Data are presented as No. (%) unless otherwise indicated. Characteristics of patients treated and not treated for IFI were compared using χ^2^ tests of independence for categorical variables and Wilcoxon rank-sum tests to compare nonnormally distributed numeric variables.

Abbreviations: AML, acute myeloid leukemia; APML, acute promyelocytic leukemia; IFI, invasive fungal infection; Q1, first quartile; Q3, third quartile; NA, not applicable; NOS, not otherwise specified; PML/RARA, promyelocytic leukemia/retinoic acid receptor alpha.

^a^Age taken at end of study period or death.

^b^Charlson comorbidity score was calculated from *International Classification of Diseases* (*ICD*), Tenth Revision discharge codes.

^c^Hematological diagnoses were classified with *ICD for Oncology* codes using the World Health Organization classification of hematopoietic and lymphoid neoplasms (4th edition, 2008).

^d^First-line chemotherapy regimens were classed as high intensity (containing anthracycline, fludarabine, or intermediate/high-dose Ara-C), intermediate intensity (containing venetoclax), low intensity (hypomethylating agent, low-dose Ara-C, or tyrosine kinase inhibitor without venetoclax), or arsenic containing.

^e^Duration of follow-up was calculated in days from study start or hematological diagnosis to study end or death.

### Antifungal Therapy Episodes

A total of 2910 antifungal prescriptions were authored for the 298 included patients during the study period, of which 860 prescriptions formed 288 therapy episodes for IFI. Of 288 antifungal therapy episodes, 115 (39.9%) did not meet consensus diagnostic criteria, meaning that 33.9% of inpatient administrations measured by DOT were given to patients without strong evidence of IFI (Table 2). The most common reasons documented for initiating these antifungal therapy episodes were indeterminate thoracic imaging findings and neutropenic colitis. Inpatient pTAFT was 21.7%.

**Table 2.** Inpatient Antifungal Use by Diagnostic Confidence Level

Characteristic	Indication, No. (Row %)			Total, No. (Column %)
	Not Meeting Diagnostic Criteria	Possible IFI	Proven/Probable IFI	
Antifungal therapy episodes				
No. of therapy episodes	115	102	71	288
% of therapy episodes	39.9	35.4	24.7	100.0
Antifungal usage metrics				
Days of therapy/1000 OBD				
All agents	54 (33.9)	71 (44.4)	35 (21.7)	160
Echinocandins	29	25	13	66 (41.3)
Triazoles	17	32	4	52 (32.6)
Polyenes	7	13	15	35 (21.7)
SXT	2	2	3	7 (4.1)
Flucytosine	0	0	1	1 (0.3)
Length of therapy	1250 (35.5)	1558 (44.2)	717 (20.3)	3525
RDD/1000 OBD	60 (32.6)	81 (44.3)	43 (23.1)	184

Abbreviations: IFI, invasive fungal infection; OBD, occupied bed-day; RDD, recommended daily dose; SXT, sulfamethoxazole-trimethoprim.

### Patients Treated for IFI

Of 298 patients, 124 (41.6%) received treatment for IFI. Sex and Charlson comorbidity score were consistent between patients who were and were not treated for IFI (Table 1). Patients who were treated for IFI had a lower median age (*P* = .0664) and were more likely to have received high-intensity first-line chemotherapy (*P* < .001) than patients who did not receive antifungal treatment, and a higher proportion of them died during the study period (43.5% vs 32.8%, *P* = .076).

### Incidence of IFI

Cumulative incidence of IFI was 3.4% proven (10/298), 6.7% proven/probable (20/298), and 23.8% proven/probable and possible (71/298). Characteristics of patients in each group are shown in Table 1. One patient had probable IFI (serially positive serum galactomannans with thoracic imaging suggestive of aspergillosis) and was not temporally linked with an antifungal therapy episode. Manual review of case notes confirmed this patient did not receive antifungal therapy and instead received palliative care. Mycological evidence of IFI observed in proven and probable cases is shown in Table 3.

**Table 3.** Mycological Evidence in Cases of Proven/Probable Invasive Fungal Infection

Infection	No.	Pathogen	Fungal Invasion of Tissue on Histology	PCR of Fixed Tissue	Fungi Isolated on Culture	Fungal Elements on Microscopy	Serum GM >0.7 U/mL	Consecutive BDG >80 pg/mL	Single BDG >80 pg/mL	PJP PCR
5 proven yeast infections	1	*Saprochaete clavata*	…	…	Blood culture	…	…	…	+	…
	1	*Saprochaete clavata*	…	…	Blood culture	…	…	+	…	…
	1	*Candida dublinensis*	…	…	Blood culture	…	…	+	…	…
	1	*Saccharomyces cerevesiae*	…	…	Blood culture and line tip culture	…	…	…	…	…
	1	*Candida tropicalis*	…	…	Drain fluid (thigh abscess)	…	…	…	+	…
5 proven mold infections	1	UID	Lung biopsy	…	…	…	…	+	…	…
	1	Mucorales sp	Lung biopsy	…	…	…	+	…	…	…
	1	*Aspergillus niger*	…	…	Pleural fluid	…	…	…	…	…
	1	UID	Tonsillar biopsy	Tonsillar biopsy	Tissue culture (tonsillar biopsy)	…	+	…	+	…
	1	*Geotrichum clavatum*	Liver biopsy	…	Tissue culture (liver biopsy)	…	…	+	…	…
10 probable IFIs	1	UID	…	…	…	BAL fluid	…	…	…	…
	1	*Aspergillus flavus*	…	…	Sputum culture	…	…	…	…	…
	1	*Aspergillus fumigatus*	…	…	Sputum culture	…	…	…	…	…
	1	*Aspergillus fumigatus*	…	…	Endotracheal aspirate	…	+	…	…	…
	1	UID	…	…	…	…	+	…	…	Low level BAL fluid
	1	…	…	…	…	…	+	…	…	…
	1	…	…	…	…	…	+	…	+	…
	2	…	…	…	…	…	+	+	…	…
	1	…	…	…	…	…	…	+	…	…

Mycological evidence presented as per European Organisation for Research and Treatment of Cancer/Mycoses Study Group Education and Research Consortium guidance for diagnosis of IFI.

Abbreviations: BAL, bronchoalveolar lavage; BDG, β-D-glucan; GM, galactomannan; IFI, invasive fungal infection; PCR, polymerase chain reaction; PJP, *Pneumocystis jirovecii*; UID, unable to identify.

All cases of possible IFI were diagnosed based on thoracic computed tomography appearances suggestive of invasive pulmonary mold infection. Fifty-three of 124 (42.7%) patients who received therapeutic antifungals never met diagnostic criteria.

### Galactomannan and β-D-Glucan

Galactomannan and β-D-glucan contributed to diagnosis of probable IFI in 6 and 3 patients, respectively (Table 3). Test positivity for galactomannan was 14/502 (2.8%) and β-D-glucan was 33/463 (7.1%). Less than half of the positive biomarker results were obtained from patients not already receiving antifungal therapy.

### Febrile Neutropenia and Antifungal Use

A total of 1164 periods of febrile neutropenia (FN) were observed during the study period, of which 541 (46.5%) lasted >72 hours. Of 298 patients, 253 (84.9%) had at least 1 FN period and 204 (68.5%) had at least 1 period lasting >72 hours (Figure 1*B*).

**Figure 2.**
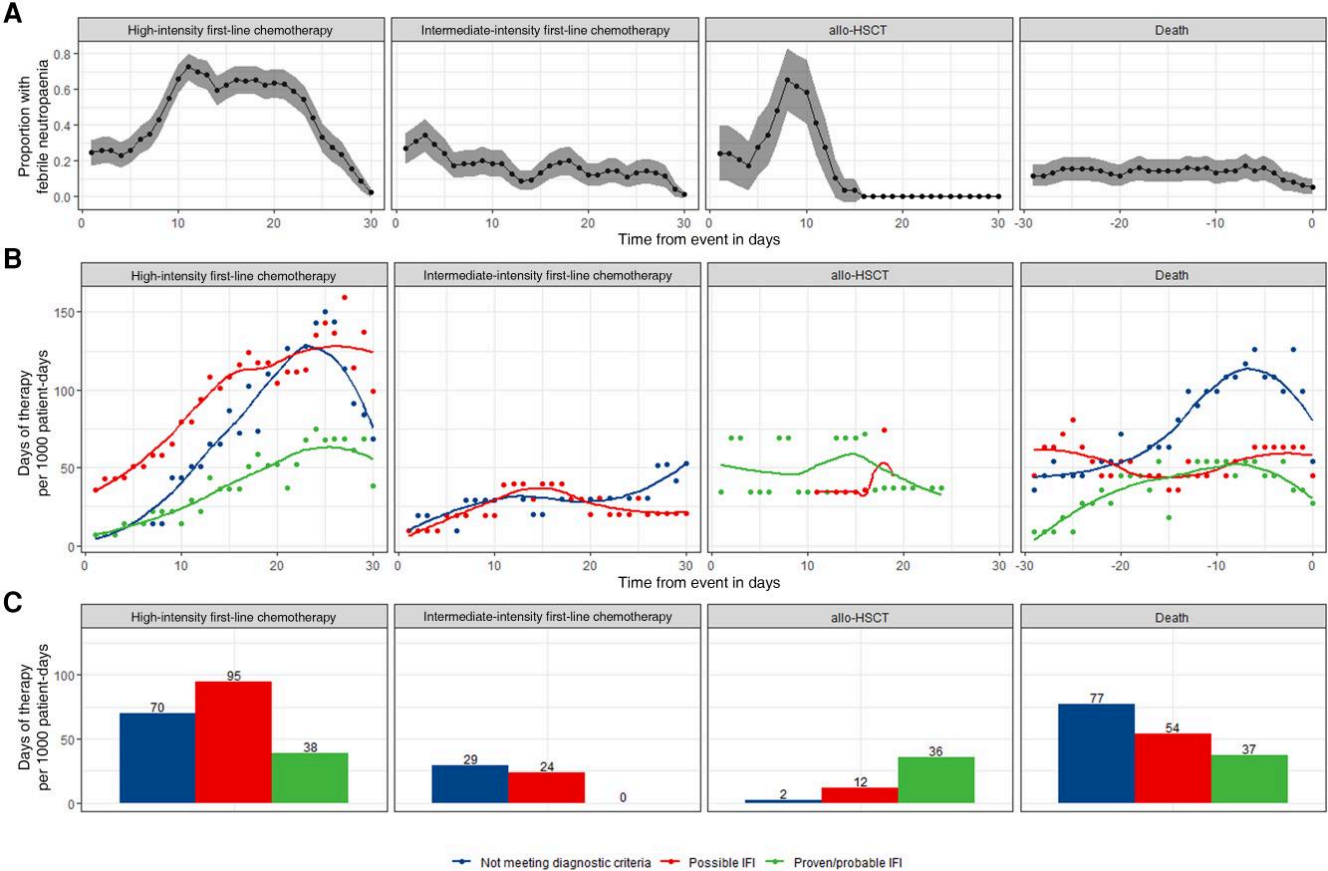
Febrile neutropenia and antifungal use. The burden of febrile neutropenia was considered alongside antifungal use (inpatient and outpatient) during three 30-day snapshots: from day 1 of first-line chemotherapy, day 0 of allogeneic hematopoietic stem cell transplantation, and in the 30 days prior to death. First-line chemotherapy regimens were classed as high (containing anthracycline, fludarabine, or intermediate/high-dose Ara-C) or intermediate (containing venetoclax) intensity. Proportion of patients with febrile neutropenia was plotted over time with 95% confidence intervals. *A*, Antifungal therapy episodes were annotated with a level of diagnostic confidence judged by European Organisation for Research and Treatment of Cancer/Mycoses Study Group criteria. DOT (days of therapy) designates the number of days that a patient receives therapeutic antifungals regardless of the dose. On days when a patient receives >1 antifungal, >1 DOT may be counted. DOT were calculated using administration and dispensing records aggregated by level of diagnostic confidence of their associated therapy episode and plotted over time with locally estimated scatterplot smoothing (*B*) and as a bar plot showing the total use during each 30-day snapshot (*C*). Number of patients and patient-days observed in each period are given in Figure 1*B*. Abbreviations: allo-HSCT, allogeneic hematopoietic stem cell transplantation; IFI, invasive fungal infection.

Most patients receiving high-intensity first-line chemotherapy or allo-HSCT developed FN in the subsequent 30 days (96.4% [134/139] and 89.7% [26/29], respectively) with FN lasting >72 hours being more common in patients receiving high-intensity chemotherapy (78.4% [109/139] vs 41.4% [12/29]). While most FN was observed in the first 14 days following allo-HSCT, a high proportion of patients who received high-intensity first-line chemotherapy had FN beyond day 14 (Figure 2*A*). A lower but substantial burden of FN was observed in the 30 days following intermediate-intensity first-line chemotherapy (60.6% [63/104 patients]). Of patients who died, 37.8% (42/111) had FN within 30 days prior to death, but the proportion observed on any given day during this period was around 15%, reducing further immediately prior to death, suggesting these data may underestimate the burden of FN at the end of life due to cessation of measurement and discharge from hospital.

Therapeutic antifungal use (inpatient and outpatient) was highest in the 30 days following high-intensity first-line chemotherapy and in the 30 days prior to death (203 and 168 DOT/1000 patient-days, respectively) with lower use in the 30 days following intermediate-intensity first-line chemotherapy and allo-HSCT (53 and 50 DOT/1000 patient-days respectively; Figure 1*B*). pTAFT was highest and therefore antifungal use most rational following allo-HSCT (71.4% [36/50]). pTAFT following high-intensity first-line chemotherapy and in the 30 days prior to death was 19.0% [39/203] and 22.1% [37/168], respectively. Antifungal use in cases not meeting diagnostic criteria rose from day 14 post–high-intensity chemotherapy onward, and in the days immediately prior to death (Figure 2*B*). Lowest pTAFT was observed following intermediate-intensity chemotherapy (0.0% [0/53]) as no patients were treated for proven/probable infection (Figure 2*C*).

### Thoracic HRCT and Initiation of Antifungal Therapy

**Figure 3.**
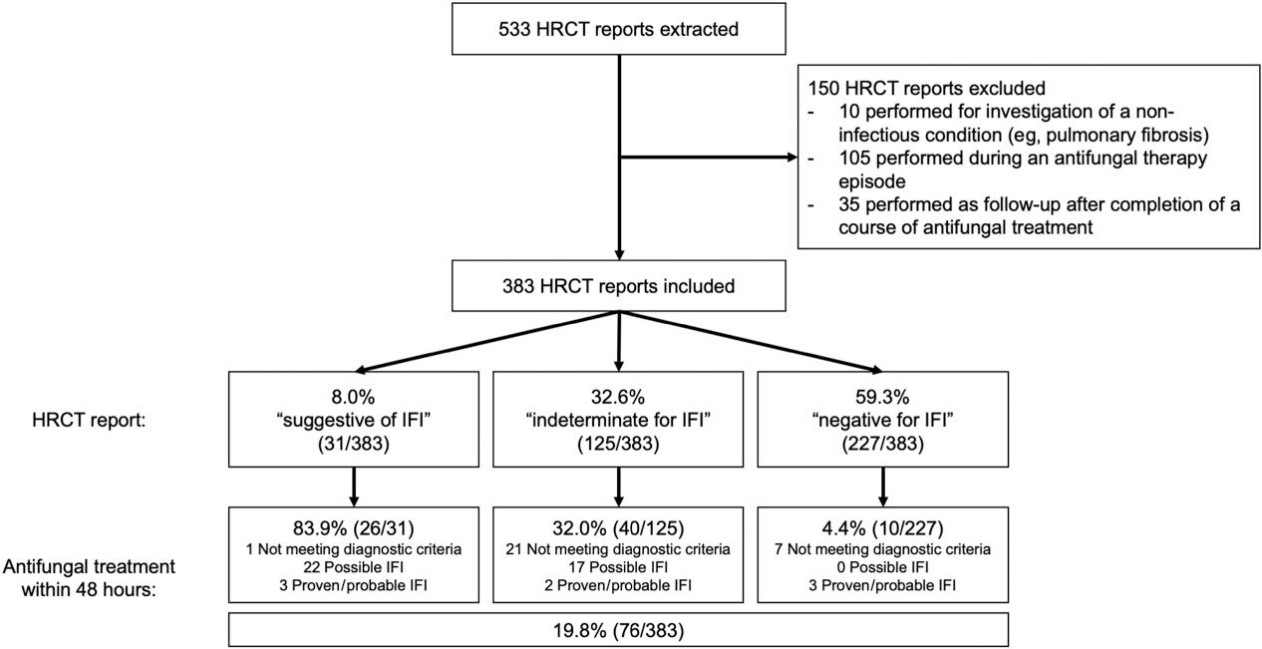
Initiation of antifungals within 48 h of high-resolution computed tomography (HRCT). HRCT reports were categorized based on the overall level of diagnostic certainty for invasive fungal infection (IFI) expressed by the reporting radiologist as previously described [16].

Five hundred thirty-three thoracic HRCT scans were performed during the study period (1.8 per patient); 150 of these were excluded from the analysis (Figure 3). The remaining 383 HRCT scans were performed for new suspicion of IFI; 31 (8.0%) reports were suggestive of IFI, 126 (32.6%) were indeterminate, and 227 (59.3%) were negative for IFI. Overall, therapeutic antifungals were started within 48 hours after 76 of 383 (19.8%) of the HRCT studies. An HRCT with an indeterminate report was almost 8 times more likely to be followed by a new antifungal therapy episode within 48 hours than a negative scan (32.0% vs 4.4%).

## DISCUSSION

We demonstrate the utility of a novel approach for determining patterns and drivers of antifungal prescribing. We derived therapy episodes from routinely collected prescription records and annotated them with consensus diagnostic criteria enabling antimicrobial use to be measured by level of diagnostic confidence. We show that, overall, the pTAFT in inpatients was 21.7%.

Compared to diagnostic criteria designed for research studies, clinicians have a lower threshold of suspicion for initiating antifungal therapy [1]. Invasive investigations (eg, bronchoscopy or biopsy) may be deemed unsafe in sicker patients. Physicians tend toward prescribing antimicrobials as a risk-reducing active choice in the face of diagnostic uncertainty [15]. This may explain the lower pTAFT observed in in patients receiving high-intensity first-line chemotherapy where prolonged neutropenic fever was common (observed in 78.4% of patients) and at the end of life where empirical antifungal treatment likely represents a last resort in the deteriorating patient [15]. Antifungal overuse may be particularly prevalent in these groups. However, although antifungal use in patients with “possible IFI” may not have been “targeted,” it would not necessarily have been inappropriate either. Framing the pTAFT metric as a percentage is helpful to describe the degree of diagnostic confidence in different situations. pTAFT immediately following allo-HSCT was high (71.4%). Allo-HSCT is only indicated in patients who have survived first-line chemotherapy and achieved remission and, therefore, these patients may be more likely to be fit to undergo aggressive investigation. In this study we present pTAFT as a novel metric for AFS teams but do not delineate specific targets, which will depend on the population studied and should be developed locally.

Incidence of proven/probable IFI was comparable to other similar published cohorts [16–18]. We demonstrate that deriving antifungal therapy episodes from EHRS prescription records facilitates epidemiologic surveillance of IFI, which is a time-consuming manual process for clinicians. Therapy episodes enable directed interrogation of relevant time periods and, once annotated with EORTC/MSG criteria, can be aggregated by patient to estimate cumulative incidence (missing only 1 case of probable IFI in this cohort). In the future, expert surveillance systems could incorporate automated annotation of antifungal therapy episodes to support continuous monitoring in at-risk groups [19].

Fungal biomarkers had a noticeably low positivity rate in our patients, 94.6% of whom received mold-active prophylaxis, which is known to reduce sensitivity of serum galactomannan [20]. Moreover, we did not employ biomarker screening. Our results may not be generalizable to settings where mold-active prophylaxis is withheld and biomarker screening is used, where a higher pTAFT might be expected. An ongoing UK-based randomized controlled trial (RCT) will compare this latter approach with mold-active prophylaxis as standard of care [21].

Abnormal thoracic imaging was the most common reason for initiating “diagnostic-driven” antifungal therapy [22]. We found that radiologists expressed a high degree of uncertainty when reporting thoracic HRCT in these patients, and indeterminate reports were associated with initiation of new antifungal therapy episodes. A recent RCT reported that abnormalities on HRCT triggered preemptive caspofungin therapy more often than fungal biomarkers, even in patients not receiving mold-active prophylaxis and undergoing twice-weekly galactomannan screening [16]. The proportion of patients who received antifungal therapy in our study (41.6%) was somewhere between the rates seen with the protocolized empirical and diagnostic-driven treatment strategies (63% and 27%, respectively) evaluated in this RCT. This implies that current “real-life” practice may involve a mixture of these 2 approaches.

This study has several limitations. First, including outpatient alongside inpatient antifungal use without overestimating overall use was challenging [23]. For example, overestimation could occur when patients were readmitted and administered inpatient doses before exhausting their dispensed outpatient supply. We therefore only included outpatient use during specific snapshot periods where each dispensing record was reviewed manually. Second, there is likely to be variation in therapeutic antifungal strategy between prescribers. Given that proven and probable IFI are rare events, we were unable to explore this in detail. Third, patients may have been hospitalized or have received antifungal therapy at other facilities, which would not have been captured by our EHRS. Finally, as a retrospective cohort study, our study is limited in its capacity to identify causation and it is not possible to infer how or why particular antifungal treatment decisions were made. It is a challenge for researchers, even in interventional studies, to determine whether a specific course of treatment was appropriate or inappropriate [24].

Instead, we developed an approach to explore patterns of prescribing at a population level. We describe a reproducible method to analyze routinely collected clinical data on antifungal therapy episodes in a widely used EHRS. We calculated the pTAFT, a new metric that will likely be useful to AFS programs. Our approach could be adapted for use in cohort studies of other infectious conditions that have consensus diagnostic criteria. Antifungal stewardship remains challenging in the absence of reliable diagnostics, particularly in unwell patients. The thoracic HRCT report is an important contributor to diagnostic uncertainty. These important insights will inform the development of new AFS interventions. For example, discretizing thoracic imaging results, either through standardized reporting frameworks or artificial intelligence image interpretation, might help safely reduce antifungal overuse [25].

## Supplementary Material

ofae094_Supplementary_Data

## Supplementary Data

Supplementary materials are available at *Open Forum Infectious Diseases* online. Consisting of data provided by the authors to benefit the reader, the posted materials are not copyedited and are the sole responsibility of the authors, so questions or comments should be addressed to the corresponding author.

## Notes

***Acknowledgments***. This study has been supported by the National Institute for Health and Care Research (NIHR) University College London Hospitals (UCLH) Biomedical Research Centre (BRC), in particular by the NIHR UCLH/University College London BRC Clinical and Research Informatics Unit. We also thank Kawsar Noor, who extracted free-text imaging reports using the CogStack platform; and Mingze Sun, who supported their compilation.

***Author contributions***. H. H., W. K. W., A. J. W., and N. S. made substantial contribution to the conception of the work. H. H., P. D.-M., S. P., and L. S. developed the data collection and statistical analysis methodology. H. H., M. S., A. J. W., and N. S. made substantial contribution to the design of the analyses. H. H. performed the analysis. H. H., M. S., S. P., A. J. W., and N. S. contributed to data interpretation. H. H. drafted the manuscript. All authors contributed to revising the manuscript critically for important intellectual content, approved the final manuscript, and are accountable for all aspects of the work.

***Previous presentations***. Initial data were presented as a research poster at the European Congress of Clinical Microbiology and Infectious Diseases 2023 and an oral presentation at the British Society for Medical Mycology Conference 2023.

***Patient consent***. This study was performed as part of routine antimicrobial stewardship activities and used routinely collected clinical data. As such, formal ethical approval and patient consent were not required. The study was registered as a service evaluation within the Department of Infectious Diseases, University College London Hospitals NHS Trust.

***Disclaimer***. Gilead Sciences had no input into the study proposal, design, data collection, or manuscript preparation.

***Financial support.*** This work was supported by Gilead Sciences, Inc. through the Gilead UK and Ireland Fellowship and Medical Grants Programme (grant number 14682 to A. J. W. and N. S.). This funding supported a research fellowship completed by H. H.
