# Supplementary material for: Patterns and Drivers of Antifungal Prescribing in Acute Leukemia: A Retrospective Cohort Study
Source: Open Forum Infect Dis. 2024 Mar 1;11(6):ofae094. doi: 10.1093/ofid/ofae094 (PMC11181189; doi:10.1093/ofid/ofae094)
Supplement: ofae094_Supplementary_Data [file ofae094_supplementary_data.docx]

Supplementary materials

1. UCLH antifungal guideline for patients with haematological malignancies

*Primary antifungal prophylaxis*

All patients receiving high- or intermediate-intensity chemotherapy or allo-HSCT received anti-mould prophylaxis

Choice of antifungal agent for prophylaxis

- 1^st^ line: Itraconazole * except:
- During treatment with venetoclax: Posaconazole
- During treatment with gemtuzumab ozogamicin OR raised LFTs (i.e. 3xULN): Ambisome® IV
- During treatment with gemtuzumab ozogamicin AND CrCl<70ml/min: Caspofungin IV

* First choice agent for prophylaxis updated to Posaconazole in Dec 2023 (after study period)

*Management of suspected invasive fungal infection (IFI)*

Choice of antifungal agent for suspected IFI

- 1st line is Caspofungin IV except:
- If liver toxicity / treatment failure^†^, switch to: Ambisome® IV

† Failure of treatment is defined as progression of clinical features (fever, deterioration of respiratory function, hypotension, haemoptysis or chest pain) after at least 7 days of adequate antifungal treatment

IV to PO switch

- If resolution of fever (<38.0°C) AND clinically improved AND patient able to tolerate oral medication, switch to oral Posaconazole to complete course

UCLH: University College London Hospital; allo-HSCT: allogeneic haematopoietic stem cell transplantation; LFTs: liver function tests; ULN: upper limit of normal; IV: intravenous; CrCl: creatinine clearance; PO: oral.
